# Malignant transformation of tailgut cyst to squamous cell carcinoma, a rare case with poor outcome

**DOI:** 10.1002/ccr3.6893

**Published:** 2023-02-05

**Authors:** Mahsa Moshtaghian, Reza Shahsiah, Fatemeh Jafari, Mahdi Aghili

**Affiliations:** ^1^ Cancer Research Center Iran Cancer Institute Imam Khomeini Hospital Complex Tehran University of Medical Sciences Tehran Iran; ^2^ Department of Pathology Tehran University of Medical Sciences Tehran Iran

**Keywords:** malignant, SCC, tailgut cyst

## Abstract

Tailgut cyst, a type of retro‐rectal cyst, is a rare condition requiring evaluation for malignant transformation. We report a case of squamous cell carcinoma arising in the retro‐rectal cyst, in a 51‐year‐old female who underwent incomplete resection of the cyst and chemo‐radiotherapy, subsequently became locally recurred and metastatic.

## Abstract 2

Malignant transformation of tailgut cysts should be detected. The treatments based on pathology include complete surgical excision with or without chemotherapy and radiotherapy. Our case had a poor outcome despite the multi‐modality treatments.

## 1 INTRODUCTION

Tailgut cysts (TGCs), a type of epidermoid cyst developing in the retro‐rectal region, are rare conditions that are often asymptomatic and require further evaluation for malignant transformation if they become symptomatic or even incidentally discovered. Symptoms can range from asymptomatic to local effects on adjacent organs or infectious processes.

Tailgut cysts may have a malignant transformation and present with different pathologies that can show different behavior and outcome. Malignant transformations are often adenocarcinomas and neuroendocrine forms. Squamous Cell Carcinoma, like what we will report in our case, is very rare and has been reported in only four patients so far.

## 2 CASE REPORT

A 51‐year‐old woman presented with pelvic pain, constipation, and fecal urgency complaints. She had no past medical history or any familial history.

In rectal examination she had a palpable rectal mass in distal part with mild tenderness in touching. The rectal lumen was intact with flat mucosa on colonoscopy, but posteriorly, the rectum seemed under external pressure.

**FIGURE 1:**
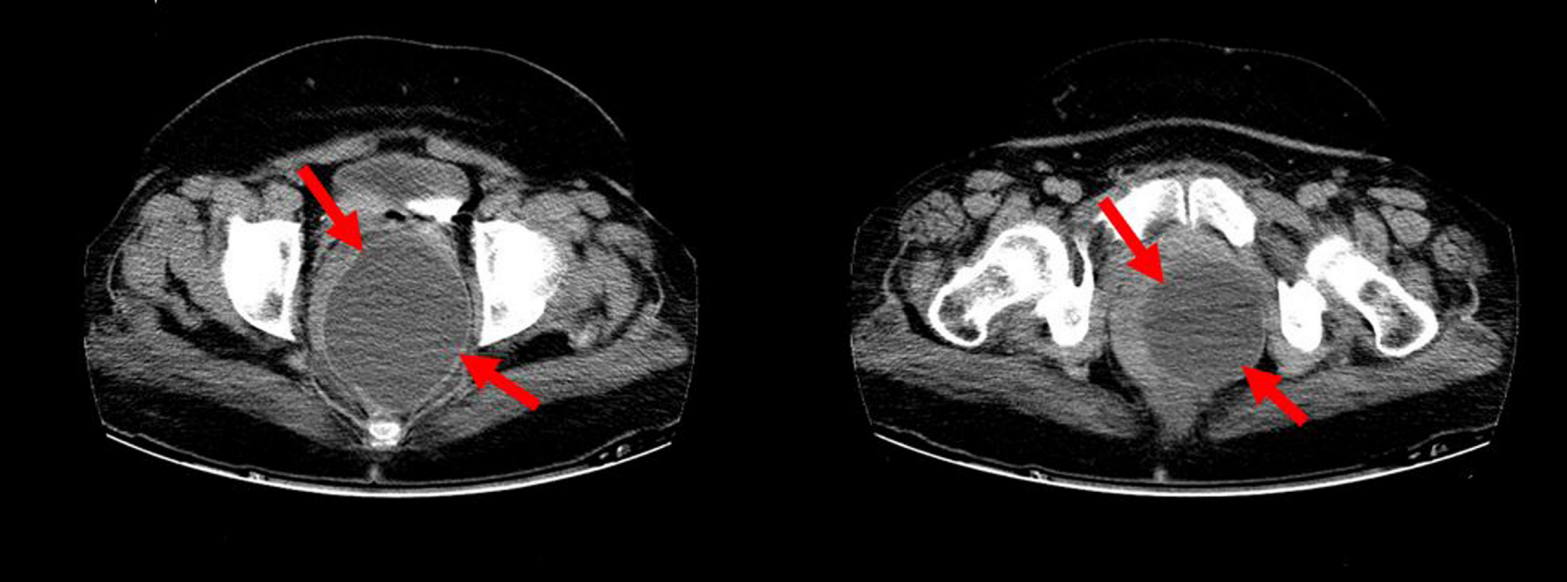
Contrast computed tomography reveals a hypodense cystic lesion measuring about 99*93 mm in the left retro‐rectal region (arrowhead) without contrast enhancement, causing a rectal deviation to the right side.

The abdominopelvic computed tomography (CT) scan revealed a hypodense cystic lesion measuring 99*93 mm in the left retro‐rectal region without contrast enhancement, causing a rectal deviation to the right side (Figure 1).

**FIGURE 2:**
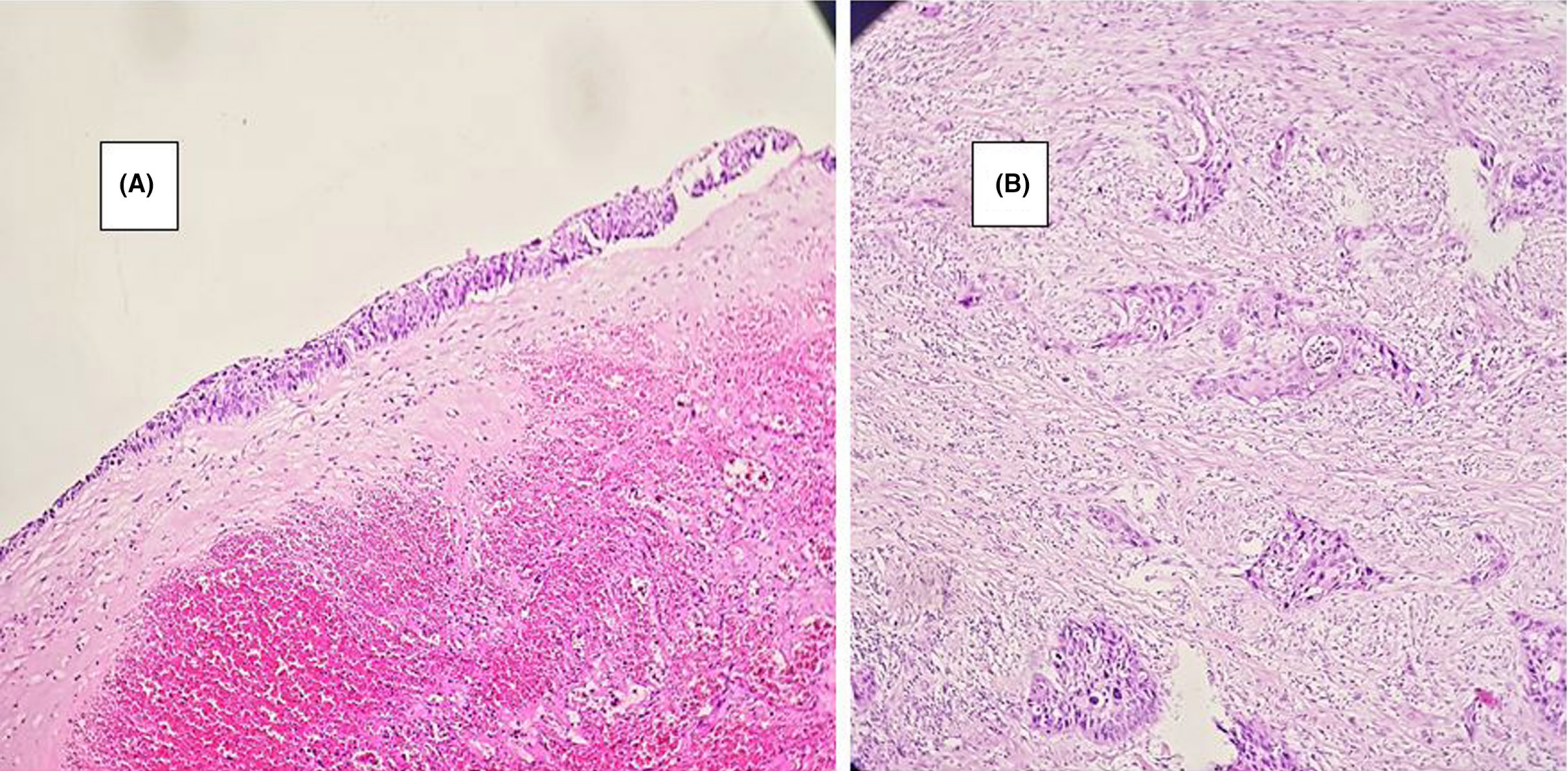
(A) Cyst wall containing smooth muscle bundles and lined by squamous and columnar epithelium (H&E, ×100). (B) The stroma is infiltrated by nests of neoplastic cells with vesicular nuclei and abundant eosinophilic cytoplasm with foci of keratin‐like material.

**FIGURE 3:**
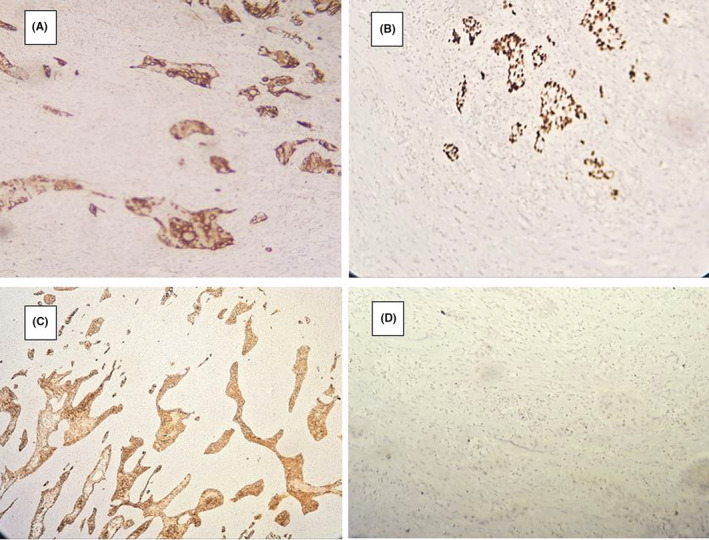
Immunohistochemical staining. (A) CK7 positive, (B) HMWCK positive, (C) P63 positive, (D) CK20 Negative.

She underwent surgical cystectomy drainage and incomplete resection of the cyst. Histopathologic examination showed a cyst wall containing smooth muscle bundles lined by squamous or columnar epithelium. The stroma was infiltrated by nests of neoplastic cells with vesicular nuclei and abundant eosinophilic cytoplasm with foci of keratin‐like material (Figure 2). In immunohistochemical staining, CK7, HMWCK, and P63 were positive, but CK20 was negative (Figure 3). These findings were compatible with squamous cell carcinoma arising on tailgut cyst.

A Post‐operative CT scan reveals no gross tumor residue. According to the multidisciplinary team's recommendation, the patient was decided to undergo radiation therapy similar to the anal canal radiation fields; 36 Gy in 20 fractions to the pelvic and inguinal lymph nodes and then up to 54 Gy to the surgical bed, concurrently with Capecitabine.

**FIGURE 4:**
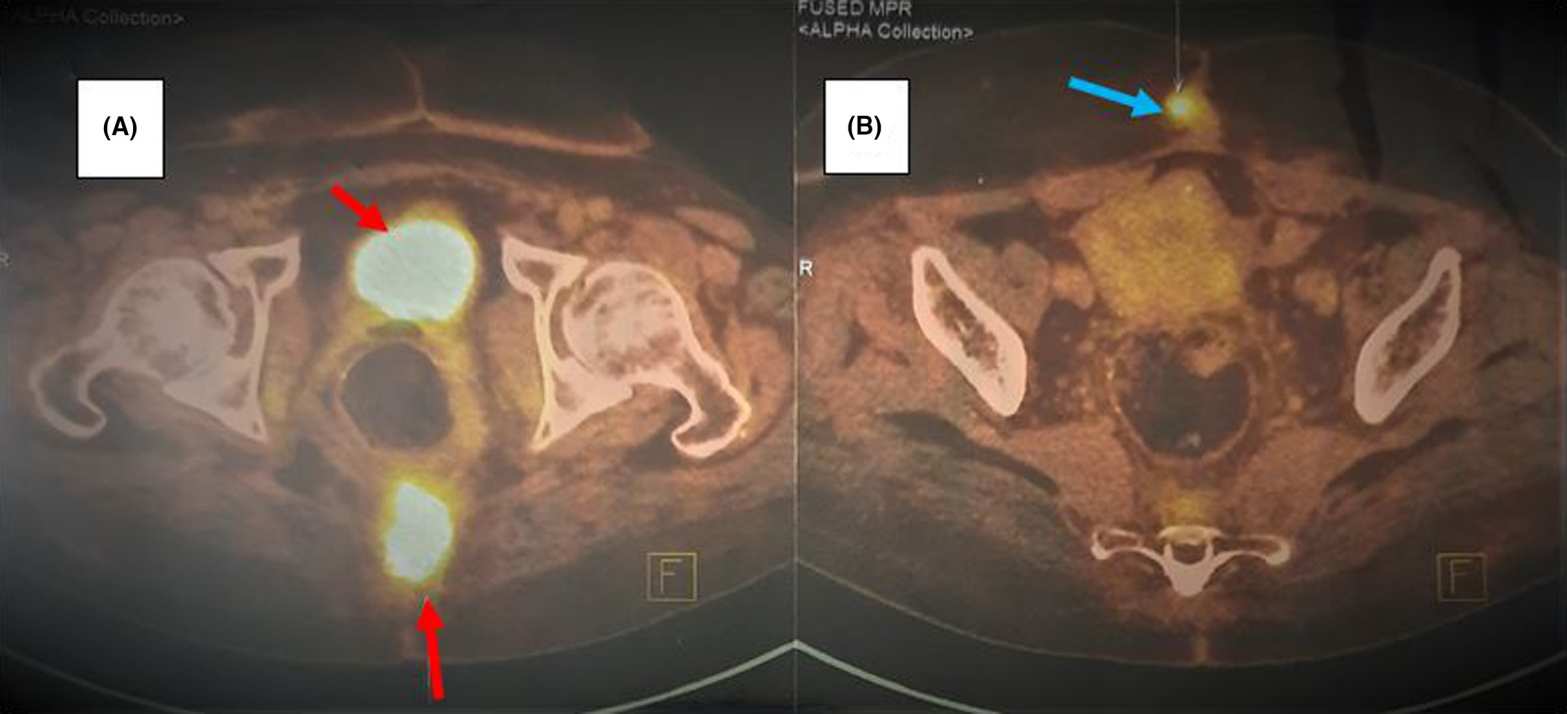
FDG‐PET/CT scan (A) There is an FDG avid (SUVmax = 17.0) soft tissue mass in the pre‐coccygeal region (arrows) measuring 3.8 × 3.0 cm. (B) There is an FDG avid (SUVmax = 6.3) soft tissue lesion in anterior pelvic wall (blue arrow) measuring 1.3 × 1.2 cm reveals metastatic lesion.

Three months after the treatment was completed, she complained of continued pelvic pain. On the pelvic MRI, there was a 23*20*28 mm multi‐loculated cystic, solid lesion in the pre‐coccygeal space in the midline posterior cavity suspicious of residual disease or recurrence. These results were confirmed by FDG‐PET/CT scan; high SUV^1^ presacral mass indicating malignancy and also a small soft tissue lesion in the anterior pelvic wall (SUV = 6.3) suspicious for metastatic disease (Figure 4).

She underwent CT guided biopsy from the per‐sacral mass, and it revealed poorly differentiated carcinoma.

Due to the multiple recurrent lesions and the presence of metastasis, she was referred for chemotherapy. There was no treatment response despite the numerous chemotherapy courses, and the disease progressed. Her pelvic pain was difficult to control by the nerve block and palliative measures. Finally, one year after the diagnosis, she, unfortunately, passed away due to the progression of metastatic abdominopelvic lesions.

## 3 DISCUSSION

Tailgut cysts (TGCs), also known as retro‐rectal cystic hamartoma (RCH) or epidermoid cysts in presacral and retro‐rectal space, are cystic lesions that occur due to developmental abnormalities in the embryonic pathways and they are the remnants of the embryonic hindgut.^2^ Tailgut cysts are very rare, have an incidence rate of 1 of 40,000–63,000 patients, and are more common in middle‐aged women.^3^, ^4^

Histologically, TGCs are multilocular cysts with squamous, mucinous, simple, pseudostratified ciliated, or transitional lining cells or may contain bundles of smooth muscle in the cyst's wall. Meningeal tissue, thyroid tissue, and glomus bodies have also been reported.^5^

The clinical manifestations of TGC can range from asymptomatic to atypical clinical symptoms because of compressive effects of mass, including constipation, rectal filling, dyschezia, infertility, dysuria, and abdominal pain. The infectious process like repeated urinary tract infection, abscess, and fistula formation may also occur with symptoms including high fever, pain, and frequent micturition.^6^, ^7^, ^8^, ^9^, ^10^, ^11^, ^12^

Differential diagnoses that should be considered for retro‐rectal masses include anal gland cyst, duplication cyst, endometriotic cyst, epidermal cyst, teratoma or dermoid cyst, sacral meningocele, pilonidal sinus, and rarely malignancies originated from colorectal or anal area or from genitourinary origins, however the location is uncommon.^5^ Although the definitive diagnosis is only possible with pathological examination, the cyst's characteristics help to differentiate between these diagnoses. For example, the pilonidal sinus is considered when pitted lesions are present on the surface. Considering other clinical symptoms such as those mentioned, helps to diagnose the origin of the cyst. Complex cysts specially those containing solid components with rapid growth raise the suspicion of malignancy.

Although FNA or other cytological biopsies have been recommended in some older articles, tissue sampling has not been recommended in more recent data due to the risk of the tumor spreading into the abdominal cavity, and the role of imaging in preoperative diagnosis has become more prominent.^13^ Tissue biopsy might be considered only for patients with high surgical risks who are not a candidate for surgery. We may have to find out about the pathology through the biopsy in this setting.^10^

CT and MRI are required to diagnose and characterize differential diagnosis and treatment strategies.^11^, ^12^ Malignant and benign lesions show different characteristics on MRI examination, especially on T2‐weighted images.^13^ Sarkar et al. believed that radiological examination could contribute to diagnosing cystic lesions in the presacral space; however, the definitive diagnosis can only be achieved by surgical exploration and histological examination.^14^

Malignant transformation in TGCs can occur but are rare, and the clear pathogenesis remains unknown. The most frequent histological types include adenocarcinoma and neuroendocrine tumors. Others include carcinoid tumors, squamous cell carcinoma, endometrioid carcinoma, adenosquamous carcinoma, transitional cell carcinoma, and sarcoma were rarely reported.^6^, ^10^, ^15^ Squamous cell carcinoma was previously reported in just four reports,^1^, ^16^, ^17^ and our case was the fifth.

Generally, the reported incidence rate of malignant tumors arising from epidermoid cysts is 0.011% –2.2%.^7^, ^8^ However, a higher rate of malignant transformation has been reported in some reviews of TGCs. Feng Liang et al. have mentioned in a review article that about 30% of reported cases of TGCs in the literature were malignant.^9^ It could be probably due to the higher chance of developing symptoms in these malignant cases. Therefore they are more likely to be diagnosed. Similarly, K. Nicoll et al. have also reported that the overall rate of neoplastic transformation was 26.6% and noted the low metastatic rate in these malignancies.^18^ We have found only 2 cases of metastasis in the literature review which were SCC and NET.^16^, ^19^

The treatment of choice for all TGCs is complete surgical excision. Additional chemotherapy or radiation therapy treatments are given if malignancy is detected based on the pathology report. The minimally invasive transanal or transrectal approach is indicated for small low‐lying, non‐infected cases because of the risk of pelvic infection in these surgical approaches.^20^ A transabdominal surgery approach is preferred in patients with suspected malignancy because of better access and visualization for a complete oncological surgery.^20^

In the case of pre‐rectal cysts, of which Tailgut cysts can be one, the possibility of malignant transformation should be considered, and in this case, surgical resection and complete evacuation of the cyst with a suitable margins may be the best measure for potential diagnosis and treatment of these lesions. In our case, complete oncological surgery was not performed, leading to early disease progression and metastasis despite chemo‐radiation therapy. This case highlights the critical role of extensive surgery, and if it is not expected to be possible, neoadjuvant treatments may facilitate this goal. If surgery is done first, it would be logical to consider adjuvant therapies. Eventually, there is no conclusive guideline or standard policy on adjuvant treatment indications in malignant tailgut cysts. Further research is needed, so treating such patients in a multidisciplinary team is reasonable.

## AUTHOR CONTRIBUTIONS

**Mahsa Moshtaghian:** Data curation; investigation; methodology; resources; software; supervision; validation; visualization; writing – original draft; writing – review and editing. **Reza Shahsiah:** Data curation; investigation; resources; writing – review and editing. **Fatemeh Jafari:** Data curation; visualization; writing – review and editing. **Mahdi Aghili:** Conceptualization; data curation; methodology; supervision; validation; visualization; writing – review and editing.

## CONFLICT OF INTEREST

The authors declare no conflict of interest.

## CONSENT

Written informed consent was obtained from the patient's next of kin to publish this report in accordance with the journal's patient consent policy.

## ETHICAL APPROVAL

None.

## Data Availability

The data that support the findings of this study are available from the corresponding author upon reasonable request.
